# Combining transcriptomics and metabolomics to identify key response genes for aluminum toxicity in the root system of *Brassica napus* L. seedlings

**DOI:** 10.1007/s00122-023-04412-z

**Published:** 2023-07-07

**Authors:** Chenyang Li, Hongsong Shi, Lu Xu, Mingli Xing, Xiaoru Wu, Yansong Bai, Mengyuan Niu, Junqi Gao, Qingyuan Zhou, Cui Cui

**Affiliations:** College of Agronomy and Biotechnology, Southwest University, Chongqing, 400715 China

## Abstract

**Key Message:**

By integrating QTL mapping, transcriptomics and metabolomics, 138 hub genes were identified in rapeseed root response to aluminum stress and mainly involved in metabolism of lipids, carbohydrates and secondary metabolites.

**Abstract:**

Aluminum (Al) toxicity has become one of the important abiotic stress factors in areas with acid soil, which hinders the absorption of water and nutrients by roots, and consequently retards the growth of crops. A deeper understanding of the stress-response mechanism of *Brassica napus* may allow us to identify the tolerance gene(s) and use this information in breeding-resistant crop varieties. In this study, a population of 138 recombinant inbred lines (RILs) was subjected to aluminum stress, and QTL (quantitative trait locus) mapping was used to preliminarily locate quantitative trait loci related to aluminum stress. Root tissues from seedlings of an aluminum-resistant (*R*) line and an aluminum-sensitive (*S*) line from the RIL population were harvested for transcriptome sequencing and metabolome determination. By combining the data on quantitative trait genes (QTGs), differentially expressed genes (DEGs), and differentially accumulated metabolites (DAMs), key candidate genes related to aluminum tolerance in rapeseed were determined. The results showed that there were 3186 QTGs in the RIL population, 14,232 DEGs and 457 DAMs in the comparison between *R* and *S* lines. Lastly, 138 hub genes were selected to have a strong positive or negative correlation with 30 important metabolites (|*R*|≥ 0.95). These genes were mainly involved in the metabolism of lipids, carbohydrates and secondary metabolites in response to Al toxicity stress. In summary, this study provides an effective method for screening key genes by combining QTLs, transcriptome sequencing and metabolomic analysis, but also lists key genes for exploring the molecular mechanism of Al tolerance in rapeseed seedling roots.

**Supplementary Information:**

The online version contains supplementary material available at 10.1007/s00122-023-04412-z.

## Introduction

Aluminum toxicity is a serious problem of crop production in acidic soils, which constitute about 50% of the world’s potentially arable lands (Kochian et al. 2005). In recent decades, the acidification of soil has become more problematic because of the excessive application of ammonium fertilizer and the increase in acid rain. Studies have proven that excess Al^3+^ content in acid soil can inhibit cellular activity in root meristem, prevent mitosis and retard root growth (Doncheva et al. 2005; Marciano et al. 2010). A healthy root system is crucial for providing anchorage, the intake of water and nutrients, sensing environmental signals and interacting with the soil microbiome (Tracy et al. 2020). Root system growth is regulated by both genetic and environmental factors (Slovak et al. 2016; Waidmann et al. 2020), and crop germplasm shows extensive genetic variation to adverse stress including aluminum tolerance. Root adaptations can enhance the plant`s resilience to Al^3+^ stress; therefore, root systems are favorable targets for potential improvement in plant growth and crop productivity (Godfray et al. 2010). The mechanisms of plant resistance to aluminum stress are complex and diverse, including secretion of organic acids, increase of rhizosphere pH, secretion of mucus, fixation of Al^3+^ in cell walls, chelation of Al^3+^ by organic acids in solution and isolation in vacuolar areas (Kochian et al. 2015). In summary, plants’ Al resistance mechanisms can be categorized according to whether they occur within or outside of the plant cell as external exclusion or internal detoxification (Wang et al. 2021a, b). With regard to the external exclusion mode of Al^3+^ toxicity, the best-studied process is that of secretion of organic acid ions such as citrate, oxalate or malate, from the root tip, which can then directly chelate external Al^3+^ and prevent toxicity (Lou et al. 2020; Ma et al. 2001; Yang et al. 2019). Most studies have focused on analyzing conventional physiological characteristics, but in-depth molecular biological analyses are lacking. Understanding the molecular mechanism underlying the regulation of root development of rapeseed to Al^3+^ stress will help us by facilitating the application of genetic engineering strategies and chemical regulation to improve aluminum tolerance in crop plants.

Quantitative trait locus (QTL) mapping is an effective method to identify aluminum tolerance genes (Bian et al. 2013). Many quantitative trait loci (QTLs) associated with Al^3+^ toxicity have been detected in a variety of crops, including wheat (Farokhzadeh et al. 2020), soybean (Li et al. 2021) and rice (Jahan et al. 2021). Consequently, QTL mapping generates a number of candidate genes due to the small population used and the low density of the genetic map (Mackay et al. 2009). High-throughput RNA sequencing (RNA-seq) technology has become a powerful tool. It offers the benefits of high sensitivity and cost-effectiveness, and it has been used to study Al^3+^ stress-induced changes in the genomes of rice (Awasthi et al. 2021), peanuts (Xiao et al. 2021) and tea (Huang et al. 2021). Similarly, transcriptome analysis can identify thousands of differentially expressed genes (DEGs) associated with Al^3+^ stress; however, it is difficult to identify all the key genes related to aluminum stress. Therefore, the integration of DEG identification with QTL linkage mapping has been considered to be the most effective way to delineate candidate genes related to complex traits. This approach has been applied to rice (Lei et al. 2020), maize (Han et al. 2022), alfalfa (Jiang et al. 2022) and grapes (Ma et al. 2022). In addition, it was also used in the determination of resistance to Al^3+^ stress during rapeseed germination (Wang et al. 2021a, b), resistance to horseshoe root disease (Kopec et al. 2021) and resistance to low temperature stress (Qin et al. 2022). Advances in transcriptome sequencing technology (RNA-seq) and metabolomics have enhanced their application in studying the response mechanism of plants to biotic and abiotic stress, and organically linked the metabolic pathways involved in differential gene expression with differentially accumulated metabolites (DAMs) (Chen et al. 2019; Meng et al. 2022; Zhao et al. 2019). Therefore, the application of multi-omics could be considered a fruitful approach for the initial discovery of key genes related to aluminum toxicity and the associated mechanisms at the molecular level.

*Brassica napus* (genome AACC, 2*n* = 38) is one of the most productive oilseed crops and an important source of protein-rich livestock feed around the world. It is mainly planted in the Yangtze River basin in China where the soil is more acidic, but aluminum toxicity has become one of the main limiting factor affecting rapeseed production in this region (Gao et al. 2021). In this study, root phenotypes, including relative root length (RRL), relative root diameter (RRD), relative root surface area (RSA), relative root volume (RRV), relative root tips (RRT) and relative dry weight (RDW), were investigated in rapeseed seedlings from the recombinant inbred line (RIL) population (ZS11 × 10D130) after exposure to Al^3+^. Next, QTLs were preliminarily located using the constructed genetic linkage map (Ye et al. 2019). Based on the gene annotation information of the reference genome of *Brassica napus*, quantitative trait genes (QTGs) were screened within mapping confidence intervals. Transcriptome sequencing (RNA-seq) was performed on an Al^3+^-resistant (*R*) line and an Al^3+^-sensitive (*S*) line from the RIL population to identify differentially expressed genes (DEGs), and genes shared by QTGs and DEGs were screened. According to the correlation between these genes and differentially accumulated metabolites (DAMs), the number of candidate genes was further narrowed. We employed a combinatorial approach of QTL mapping in a RIL population, RNA-sequence and metabolomics analysis of *R* and *S* lines to identify the key candidate genes governing root traits in rapeseed seedlings exposed to Al^3+^ stress.

## Materials and methods

### Experimental material

The RIL containing 138 lines and its parents were used for QTL mapping, and the Al^3+^-resistant line 27,034 (*R*) and the Al^3+^-sensitive line 27,143 (*S*) derived from the RIL population were used for RNA-seq, metabolomic analysis and physiological indicators determination. The RIL population came from a cross between 10D130 and Zhongshuang11 (ZS11) by the single-grain transmission method for seven consecutive generations. The 10D130 strain is a high-generation, Al^3+^-sensitive inbred line selected from interspecific hybrids of *Brassica juncea* and *Brassica oleracea* by the Chongqing Engineering Research Center, while ZS11 is an Al^3+^-resistant, conventional, high-quality rapeseed variety selected by the Chinese Academy of Agricultural Sciences. All seeds were provided by the Chongqing Engineering Research Center.

### Experimental treatments and phenotypic evaluation

Full and uniform seeds selected from 138 lines of an RIL population were surface-sterilized, rinsed with deionized water and germinated in vermiculite medium. When the seedlings showed uniform growth with 2–3 true leaves, they were transferred to a 45-hole foam seedling tray (60 × 34 × 3 cm) and placed in a plastic tray (67 × 41 × 15 cm) for hydroponic culture in 1/2 Hoagland’s nutrient solution, in a climate-controlled chamber at 25 °C for 16 h during the day, and 20 °C for 8 h at night until four true leaves developed. The seedlings were subjected to Al stress in 1/2 Hoagland’s nutrient solution with 2.40 mM AlCl_3_·6H_2_O (pH 4.0) (Wu et al. 2022); 1/2 Hoagland’s nutrient solution without Al^3+^ was used as control. The treatment solutions were replaced every four days. After 15 days of treatment, the root systems were analyzed using the LD-WinRhizo scope (Regent Instruments, QC, Canada) to measure RRL, RRD, RRV, RSA, RRTs, and RDW. Each test was conducted with three replicates. In addition, mixed root samples from each biological replicate of the *R* line were collected into thirty 1.5 ml microfuge tubes, each containing 100 mg of root sample, quickly frozen in liquid nitrogen and stored at − 80 °C for transcriptome sequencing, identification of metabolites, determination of physiological indices and qRT-PCR. The same method was used to collect root samples for the *S* line. The control and treated samples of the *R* and *S* lines were labeled *R*_CK_ and *S*_CK_, and *R*_T_ and *S*_T_, respectively.

### Determination of physiological parameters in roots

The activity of superoxide dismutase (SOD) was determined by xanthine oxidase cytochrome C. The activity of peroxidase (POD) was measured by the guaiacol method, and catalase (CAT) activity was determined by an ultraviolet absorption method. Soluble proteins (SP) were analyzed according to Sedmak and Grossberg (1977). The content of malondialdehyde (MDA) was determined by the thiobarbituric acid (TBA) reaction method. The content of Al^3+^ ions in the roots was measured by the method of Wright et al. (1987). The contents of SOD, POD, CAT, SP, MDA and Al^3+^ were assayed using kits according to manufacturer’s instructions (Suzhou Grace Biotechnology Co., Ltd, Suzhou, China). All measurements were performed in triplicate, and means ± SD were calculated.

### QTL localization via interval gene (QTGs) screening

Based on a genetic linkage map constructed earlier in our laboratory (Ye et al. 2019), Map QTL6.0 software was used to detect QTL sites related to RRL, RRD, RRV, RSA, RRT and RDW. The logarithm of the odds (LOD) threshold for a significant QTL was calculated with 1000 permutations at a significance level of *p* = 0.05. The confidence interval for each QTL was defined by LOD ≥ 2 from the peak position. QTLs were assigned names by adding the prefix of the trait abbreviations, and their locations, including the chromosome number, as determined according to McCouch et al. (1997). If more than one QTL was detected on the same chromosome for a trait, the QTLs were consecutively numbered. MapChart 2.5 software was used to depict the QTL positions.

### RNA sequencing (RNA-seq) and DEGs determination

The root samples of *R* and *S* lines from controls and Al^3+^ stress treatments were sent to Biomarker Technologies Co., Ltd (Beijing, China) for RNA extraction, library construction, and transcriptome sequencing on the Illumina sequencing platform. After removing the 3’-adapter, low-quality sequences and clean reads were aligned to the *Brassica napus* reference genome (https://www.genoscope.cns.fr/brassicanapus/cgi-bin/gbrowse/colza/) using HISAT2 software (http://ccb.jhu.edu/software/hisat2/index.shtml). The read count value was determined by HTSeq (https://htseq.readthedocs.io/en/release_0.11.1/). Fragments per kilobase million (FPKM) values were calculated to estimate gene expression levels. DESeq2 was used for differential expression analysis between the two groups based on *p* ≤ 0.01 and |log2 fold change|≥ 2. Enrichment analysis used the Kyoto Encyclopedia of Genes and Genomes (KEGG) pathway tool. TBtools software (Chen et al. 2020) was used to draw the Venn diagram and candidate gene expression heatmap.

### Metabolite profiling

Twelve root samples from the *R* and *S* lines (T, CK) were used for untargeted metabolites based on ultra-high performance liquid chromatography-quadrupole time-of-flight mass spectrometry (UPLC-Q-TOF/MS) (Biomarker Technologies Co., Ltd.). UPLC-Q-TOF/MS analyses were performed with an Acquisition I-Class plus tandem Xevo G2-XS QToF (Waters, Milford, USA). An Acquity UPLC HSS T3 column (Waters, Milford, USA) was used in this study. The samples were analyzed in both the positive and negative ion modes. Raw mass-spec data were collected using MassLynx software (version 4.2, Waters, Milford, USA), processed and analyzed by the Progenesis QI software (Waters, Milford, USA). The MetLin database (Waters, Milford, USA) and Biomark’s self-built database were used for peak annotation and identification. Simca-P software (version 14.1, Umetrics, Umeå, Sweden) was used for principal component analysis (PCA).

### Candidate gene predication

QTL intervals were aligned to the *Brassica napus* reference genome (http://www.genoscope.cns.fr/brassicanapus/cgi-bin/gbrowse/colza/). Based on the physical positions of the flanking markers, the genes located within the QTL confidence interval were extracted (QTGs). The R language Hmisc package function (Harrell 2022) was used to determine the correlations of DEGs screened after combining with QTL and transcriptome data. The prediction of candidate genes was based on two conditions: whether the expression trends of the QTL confidence interval genes (QTGs) were different between *R*_T_ vs. *S*_T_ and *R*_CK_ vs. *S*_CK_, and whether the expression trends of *R*_T_ vs. *R*_CK_ and *S*_T_ vs. *S*_CK_ were significantly different. Genes and metabolites with Pearson's correlation coefficients, |*R*|≥ 0.95 and *p* ≤ 0.01, were used to establish the related network, which was visualized using Cytoscape 3.8.0 software (Shannon et al. 2003).

### Validation of candidate genes by qRT–PCR

Quantitative RT-PCR was performed on a BioRad CFX96 real-time system using a kit from Labgic Biotechnology Co., Ltd (Beijing, China). The primer pairs were designed by qPrimerDB v1.2 (https://biodb.swu.edu.cn/qprimerdb/) and synthesized by Sangon Biotech (Shanghai, China) (Table S1). The reaction conditions were as follows: 95 °C for 5 min, followed by 40 cycles of 95 °C for 10 s, 56 °C for 30 s and 72 °C for 30 s. *BraActin7* was used as an internal control. The 2^−ΔΔCt^ method was used to calculate the normalized expression of target genes. Three biological replicates per sample were analyzed for expression levels.

### Data processing and analysis

SPSS 22.0 (SPSS Inc., Chicago, USA) was used for statistical testing of phenotypic data from the RIL population. Statistical analysis used DPS 6.0 (Analytical Software, Hangzhou, Zhejiang, China).

## Results

### Root growth of parents and RIL populations under Al^3+^ stress

As shown in Table 1, except for RRD and RDW, the root-related traits of the RIL population were inhibited by stress from 2.40 µM AlCl_3_·6H_2_O. In addition, the RRD in the *S* line was 1.017, which was less than the 1.284 of the *R* line. The other root-related traits in the *S* line ranged from 0.343 to 0.487, while those in the *R* line ranged from 0.651 to 0.906. This indicated that the tolerance of *S* and *R* lines to aluminum stress was significantly different from each other; the root growth in the *S* line was more severely inhibited than that of the *R* line after exposure to Al stress.

**Table 1:** Distribution of root-related traits in rapeseed RIL and the parent strains under aluminum stress

Relative	Parents		RIL population					
Trait	ZS11	10D130	Mean	Range	SD	CV (%)	*R* line	*S* line
RRL	0.667	0.371	0.481	0.713	0.128	26.561	0.755	0.456
RSA	0.687	0.305	0.514	0.674	0.13	25.307	0.906	0.323
RRD	1.375	0.901	1.135	0.997	0.142	12.534	1.284	1.017
RRV	0.592	0.418	0.59	0.853	0.153	25.795	0.867	0.487
RRT	0.741	0.426	0.558	1.718	0.263	46.374	0.651	0.379
RDW	1.261	0.753	1.092	2.001	0.386	35.398	0.897	0.369

**Fig. 1:**
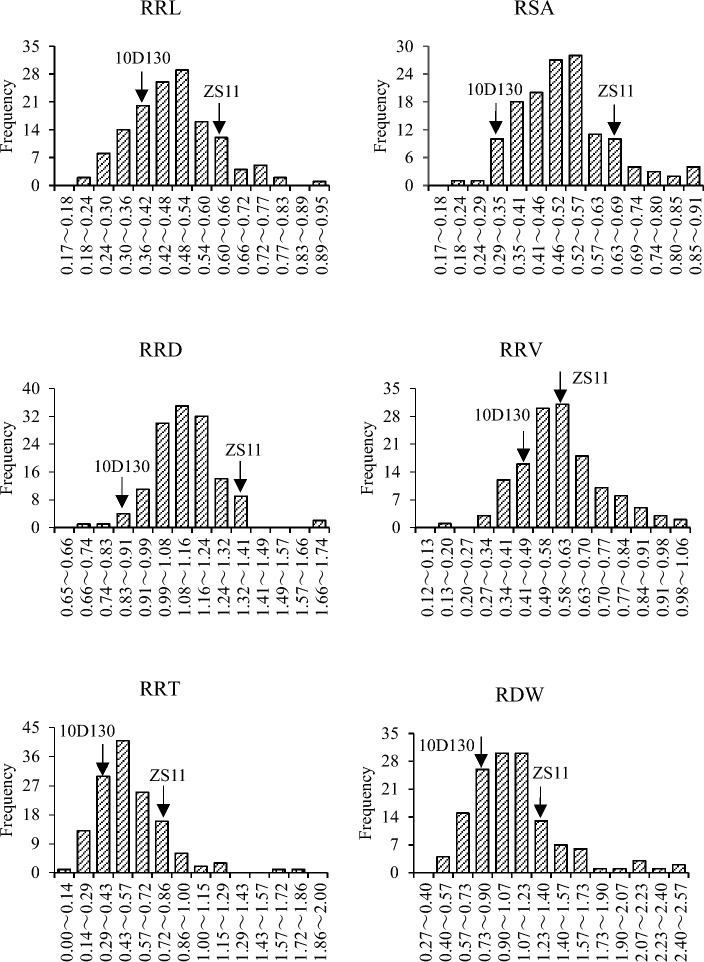
Frequency distribution of root-related traits in RIL lines under aluminum stress

Frequency analysis showed that all six traits had a continuous distribution with extensive genetic variation (Fig. 1), which was in accordance with quantitative traits and was suitable for QTL mapping.

### QTL linkage mapping of root phenotypes

**Fig. 2:**
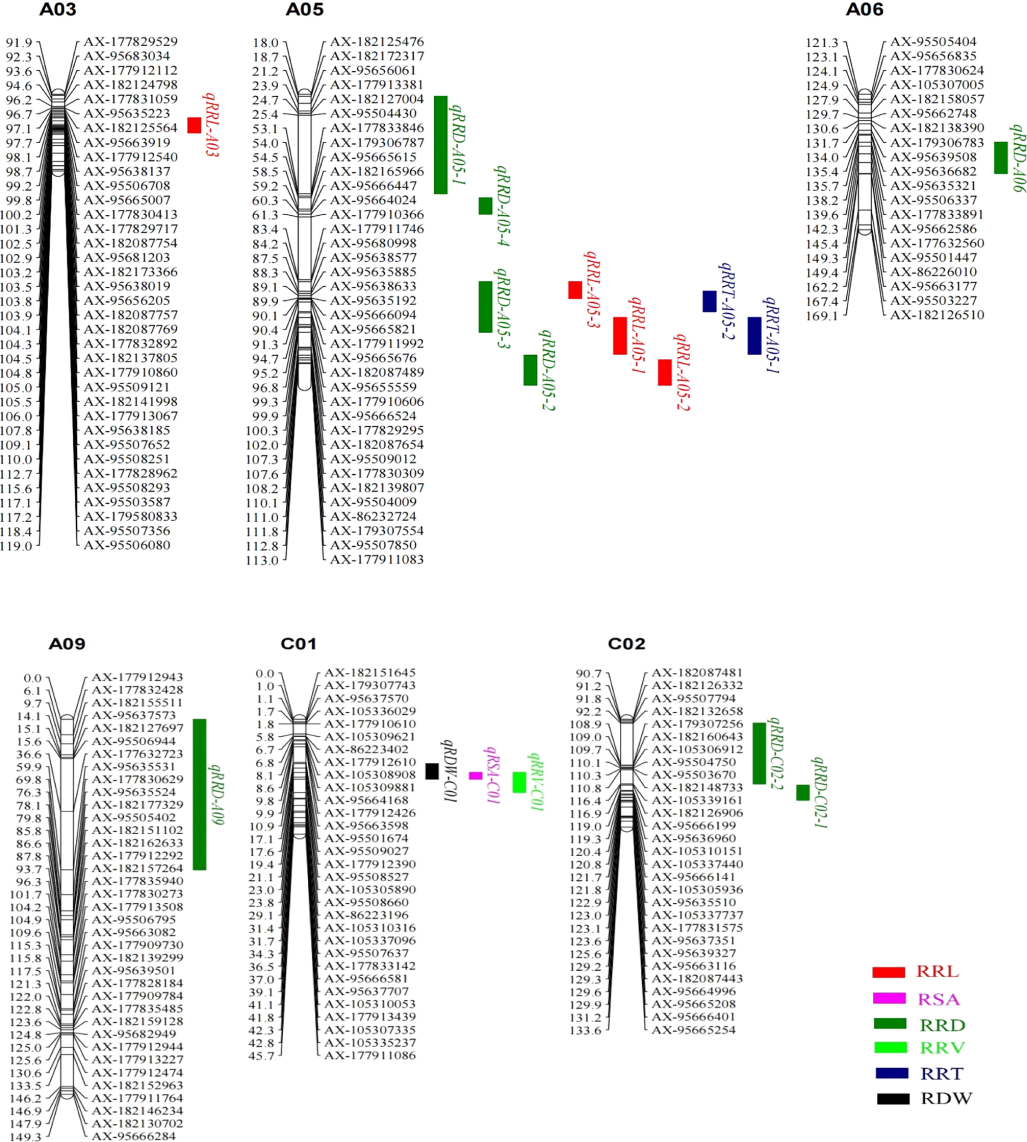
Distribution of QTLs related to aluminum tolerance on the chromosomes of rapeseed

Genotype analysis of the RIL population was conducted based on 5058 SNP markers (Table S2). In total, 17 QTLs significantly associated with aluminum stress were identified, including 4 related to RRL, 8 to RRD, 2 to RRT, 1 to RSA, 1 to RRV and 1 to RDW (Fig. 2). These QTLs explained 7.00–14.50% of the phenotypic variance, and LOD values ranged from 2.19 to 4.70 (Table 2). According to the gene annotation information of the reference genome of *Brassica napus*, 881, 57, 2679, 102, 526 and 72 candidate genes (QTGs) were screened from QTLs of the corresponding characters, respectively (Table S3).

**Table 2:** QTL mapping of root-related traits in rapeseed seedlings under aluminum stress

Trait	QTL	Marker interval	LOD value	PVE (%)	Additive effect	Confidence interval (cM)
RRL	*qRRL-A03*	AX-177830413-AX-182141998	2.40	7.7	−0.0363	100.169–105.491
	*qRRL-A05-1*	AX-95655559-AX-86232732	2.53	8.1	0.0376	96.788–109.81
	*qRRL-A05-2*	AX-179307554-AX-177911837	2.21	7.1	0.0350	111.751–120.701
	*qRRL-A05-3*	AX-95680998-AX-95666094	2.19	7.0	0.0350	84.151–90.139
RSA	*qRSA-C01*	AX-95508527-AX-95508660	2.06	6.6	0.0341	21.103–23.759
RRD	*qRRD-A05-1*	AX-182172317-AX-177833846	4.70	14.5	−0.0555	18.653–53.138
	*qRRD-A05-2*	AX-95504009-AX-177911837	3.19	10.1	−0.0464	110.146–120.701
	*qRRD-A05-3*	AX-95680998-AX-182087654	2.52	8.1	−0.0416	84.151–102.037
	*qRRD-A05-4*	AX-95665615-AX-95664024	2.25	7.2	−0.0398	54.509–60.309
	*qRRD-A06*	AX-95506337-AX-95501447	2.45	7.8	0.0415	138.207–149.348
	*qRRD-A09*	AX-177912943-AX-95635531	3.41	10.7	0.0645	0.000–59.898
	*qRRD-C02-1*	AX-179307147-AX-95635510	2.41	7.7	−0.0395	116.901–122.901
	*qRRD-C02-2*	AX-182132658-AX-105339161	2.33	7.5	−0.0397	92.161–116.354
RRV	*qRRV-C01*	AX-95508527-AX-86223196	2.27	7.3	0.0419	21.103–29.133
RRT	*qRRT-A05-1*	AX-95655559-AX-86232732	2.76	8.8	0.0781	96.788–109.81
	*qRRT-A05-2*	AX-95638577-AX-95665676	2.25	7.2	0.0712	87.45–94.748
RDW	*qRDW-C01*	AX-95509027-AX-95508660	2.15	6.9	0.1125	17.626–23.759

### Transcriptome differences between *R* and *S* lines under aluminum stress

**Fig. 3:**
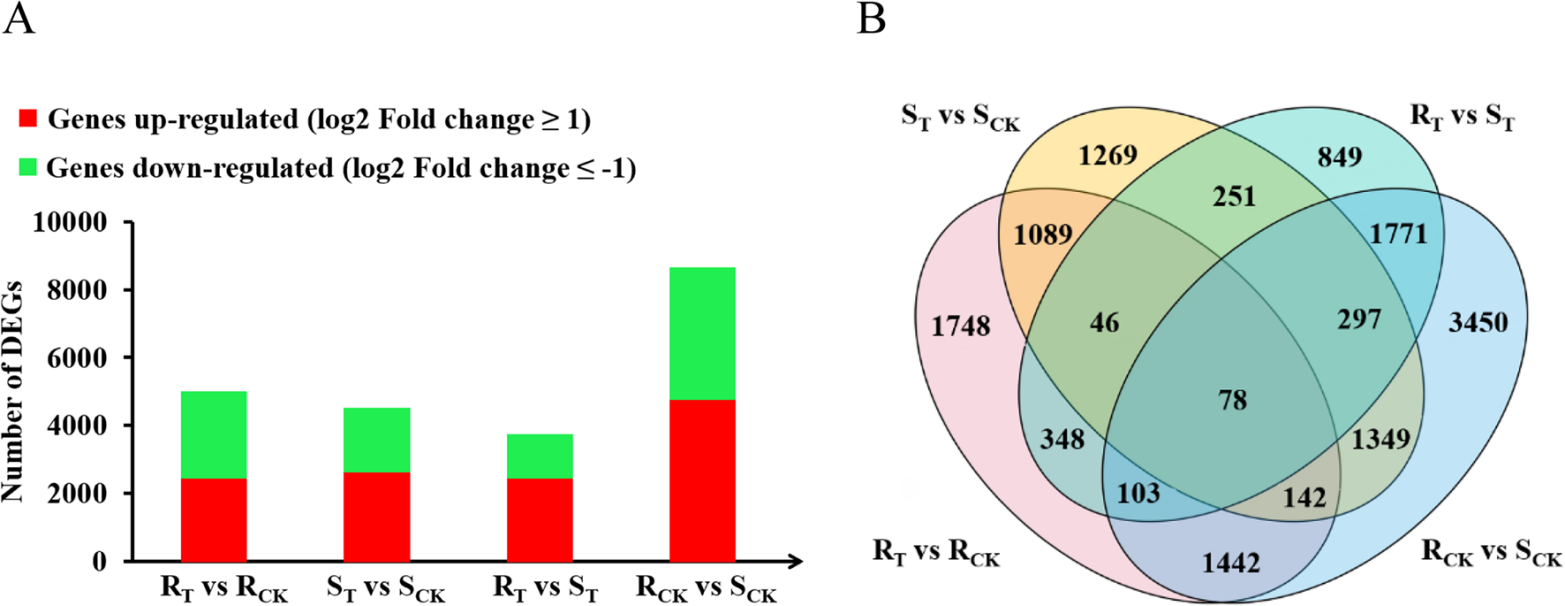
Summary of gene responses of *R* and *S* lines under aluminum stress. **A** Number of DEGs in each group; **B** Venn diagram of DEGs from each group

After filtering out low-quality reads and adapter sequences, a total of 30.24 Gb of clean data was obtained. The Q30 base percentage was at least 92.69% and 80.71–86.53% of reads could be mapped to the reference genome of *Brassica napus*, and 77.70–83.38% were unique (Table S4). As shown in Fig. 3A and B, 8945 DEGs were obtained by comparing *R*_CK_ with *S*_CK_. The number of up-regulated DEGs (4023, 45.0%) was less than that of down-regulated DEGs (4922, 55.0%). There were 5114 DEGs detected in *R*_T_ vs. *R*_CK_ and 4652 DEGs in *S*_T_ vs. *S*_CK_. Of the 5114 DEGs in the *R* line, 2634 (51.5%) were up-regulated and 2480 (48.5%) were down-regulated. The 4652 DEGs of the *S* line included 2719 (58.4%) up-regulated genes and 1933 (41.6%) down-regulated genes. In the case of *R*_T_ vs. *S*_T_, 3935 DEGs were identified including 2564 (65.2%) up-regulated genes and 1371 (34.8%) down-regulated genes. In addition, there were 1748, 1269, 849 and 3450 unique DEGs in *R*_T_ vs. *R*_CK_, *S*_T_ vs. *S*_CK_, *R*_T_ vs. *S*_T_, *R*_CK_ vs. *S*_CK_, respectively, and 78 DEGs were shared between *R* and *S* lines.

### Metabolomic analysis of *R* and *S* lines under aluminum stress

**Fig. 4:**
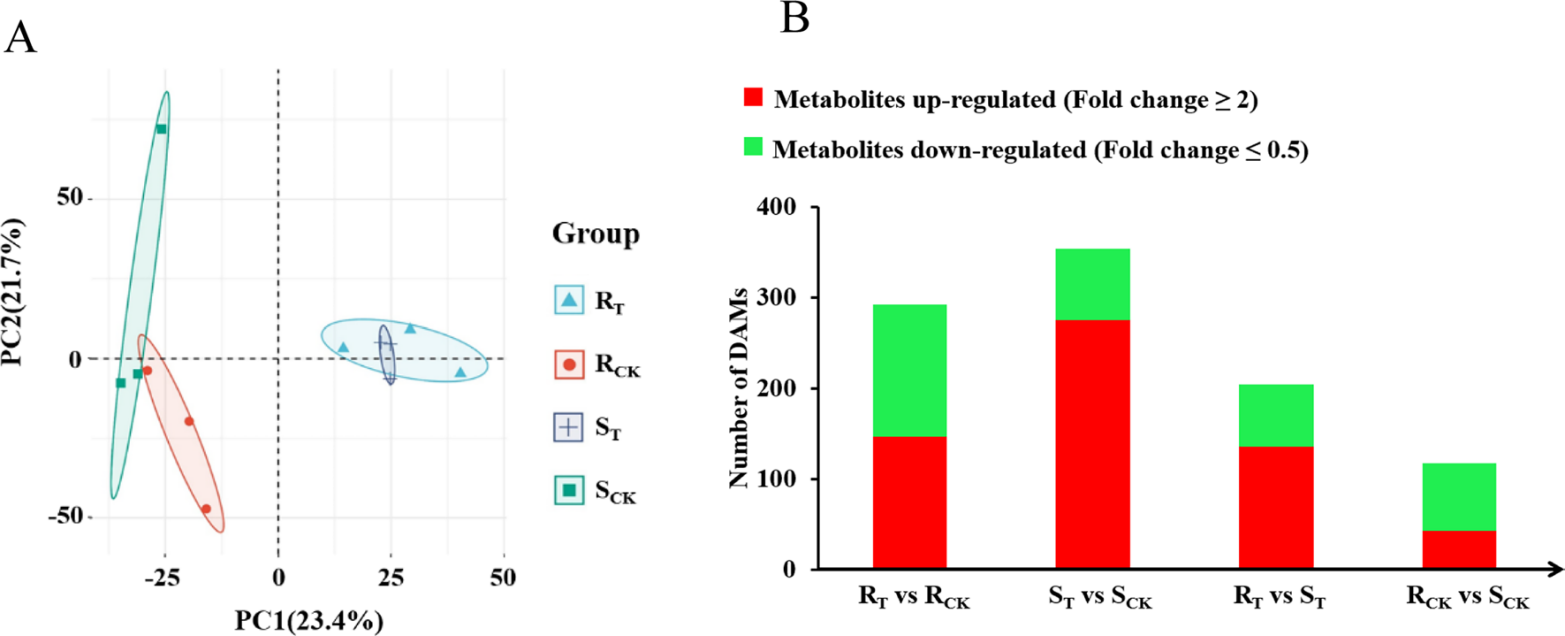
Summary of metabolome responses of *R* and *S* lines under aluminum stress. **A** PCA diagram of metabolites of *R* and *S* lines. **B** Numbers of significantly up-regulated and down-regulated metabolites in each group

Three repetitions were conducted for each treatment in the metabolic analysis to ensure the reliability of the experiment. According to PCA, the first PC (PC1) and the second PC (PC2) explained 23.4% and 21.7% of the variation, respectively, and there was an obvious separation between the control and treated samples of the *R* and *S* lines (Fig. 4A). A total of 3127 differentially accumulated metabolites (DAMs) were detected, including lipids, amino acids and their derivatives, alkaloids, carbohydrates, organic acids, terpenes, coenzymes, nucleotides and derivatives, flavonoids, alcohols, lignin and coumarins, aldehydes, phenolics and ethers (Table S5). In *R*_T_ vs. *R*_CK_, there were 292 DAMs (147 up-regulated and 145 down-regulated), 353 in S_T_ vs. *S*_CK_ (276 up-regulated and 77 down-regulated), 204 in *R*_T_ vs. *S*_T_ (136 up-regulated and 68 down-regulated) and 117 in *R*_CK_ vs. *S*_CK_, (43 up-regulated and 74 down-regulated) (Fig. 4B). After removing the DAMs with similar change trends of *R*_T_ vs. *R*_CK_ and *S*_T_ vs. *S*_CK_, the remaining 457 metabolites were considered to be related to aluminum stress (Table S6).

### Hub genes identified by integrating QTGs, DEGs and DAMs

**Fig. 5:**
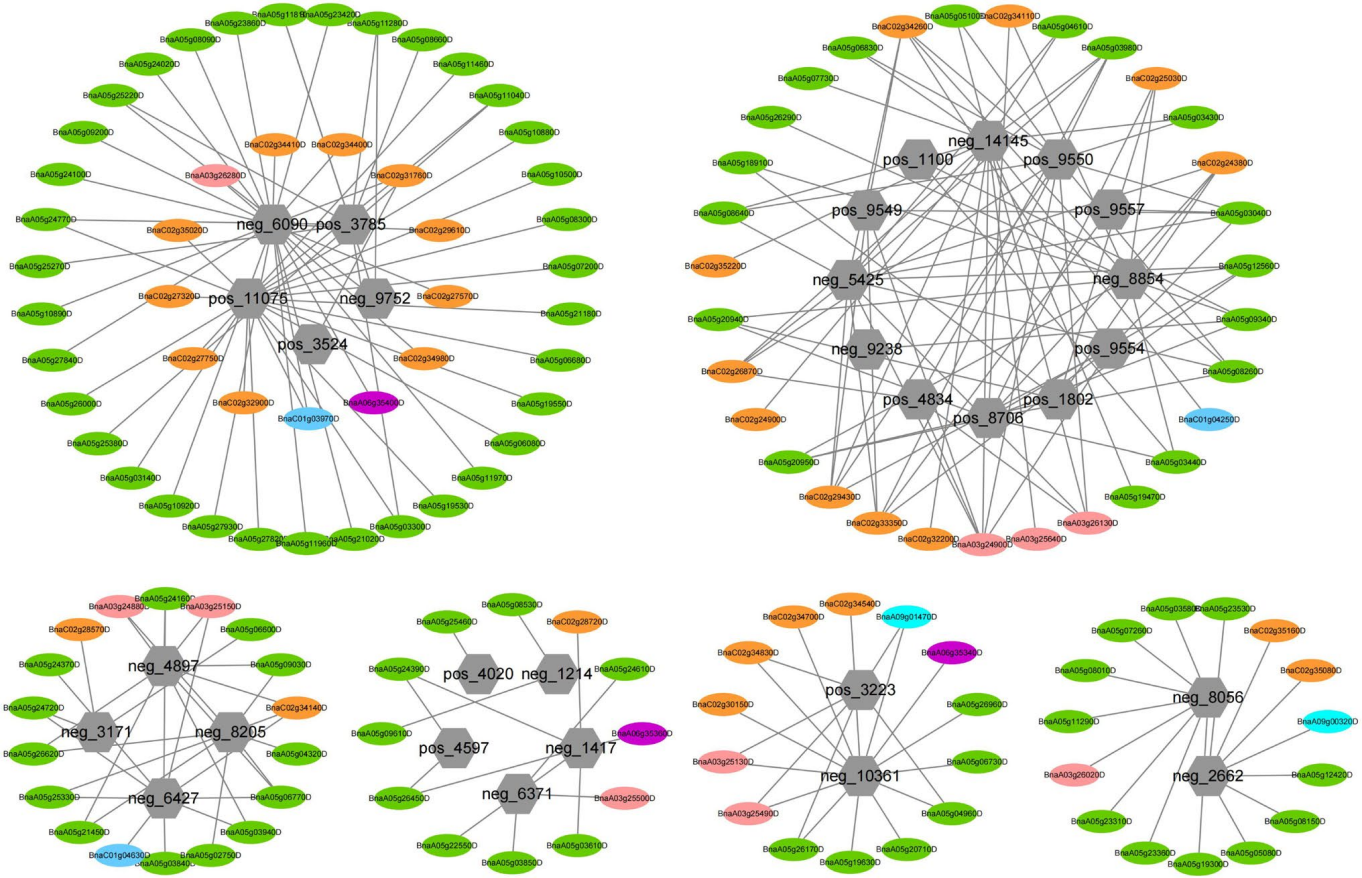
Interaction network diagram of 138 genes and 30 metabolites. Hexagons represent candidate metabolites and ellipses represent candidate genes. The chromosomes are color-coded as follows: pink A03, green A05, purple A06, turquoise A09, baby blue C01 and orange C02. The metabolites are in gray

A total of 3186 QTGs were integrated with DEGs screened by transcriptome sequencing to eliminate genes with non-significant differential expression. Based on the significant difference in QTGs expression between *R*_T_ vs *S*_T_ and *R*_CK_ vs. *S*_CK_, or *R*_T_ vs. *R*_CK_ and *S*_T_ vs. *S*_CK_, 372 candidate genes related to aluminum stress were detected (Table S7). Overall, the comparison of QTGs and DEGs further validated the reliability of the candidate genes and helped us mine key genes. Next, we conducted a joint screening of 372 DEGs and 457 DAMs to understand the mechanism underlying the response of rapeseed root to aluminum stress and establish the related network (|*R*|≥ 0.95, *p* ≤ 0.01). Subsequently, the correlation analysis showed that 138 hub genes exhibited strong positive or negative correlations with 30 important metabolites (14 in the positive mode, 16 in the negative mode) (Fig. 5).

**Fig. 6:**
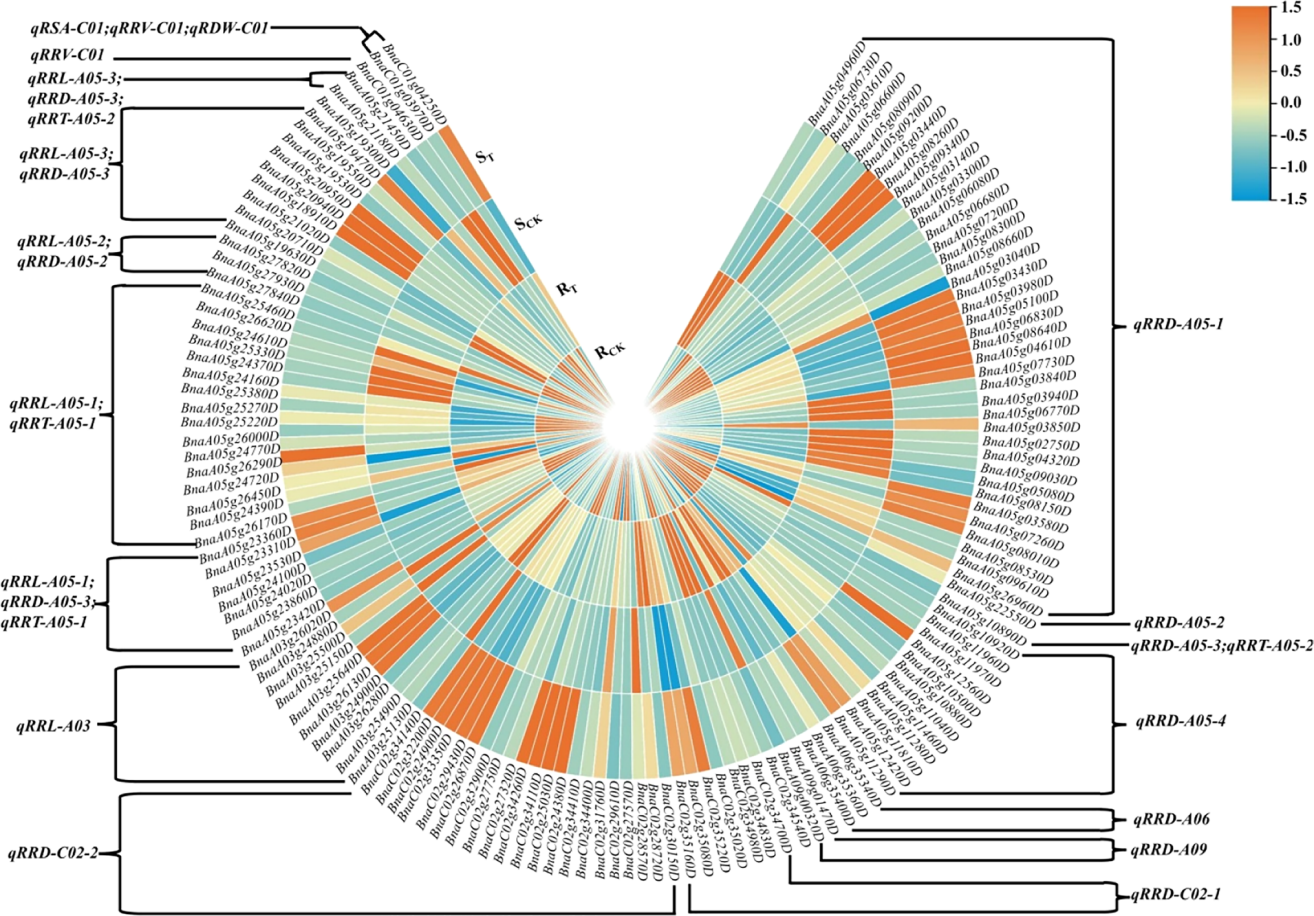
Distribution heatmap of 138 genes in QTLs

As shown in Fig. 6, 138 hub genes were identified within the confidence interval of 17 QTLs, and the number of genes in each QTL interval ranged from 2 to 38. Among the 138 genes, there were 109 key genes related to RRD, followed by 48 related to RRL, 26 to RRT, and 3 key genes to RRV. There were two key genes related to RSA and RDW, and some genes were associated with two or more traits.

### Functional analysis of hub genes and metabolites

**Fig. 7:**
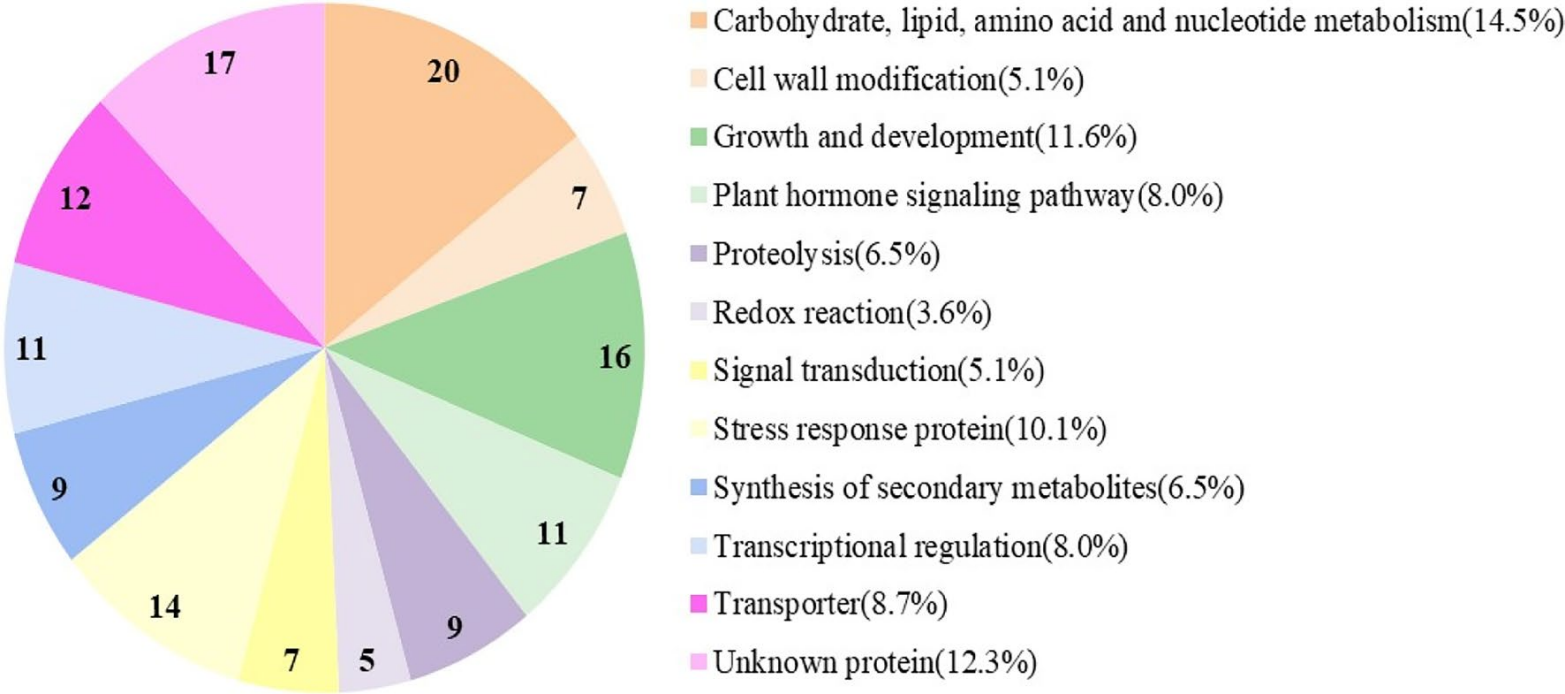
Functional classification of the 138 key genes

The 138 hub genes were aligned by BLASTP search for sequences homologous to *Arabidopsis thaliana* and divided into twelve categories according to their functions (Fig. 7, Table S8). Specifically, there were seven genes related to cell wall modification, 16 to growth and development, 11 to hormone signaling pathways, 9 to protein hydrolysis, 5 to redox reactions, 7 to signal transduction, 14 to stress reactions, 9 to synthesis of secondary metabolites, 11 to transcriptional regulation, 12 to transport proteins and 20 genes to carbohydrate and lipid metabolism. In addition, there were 17 genes encoding unknown functional proteins.

**Fig. 8:**
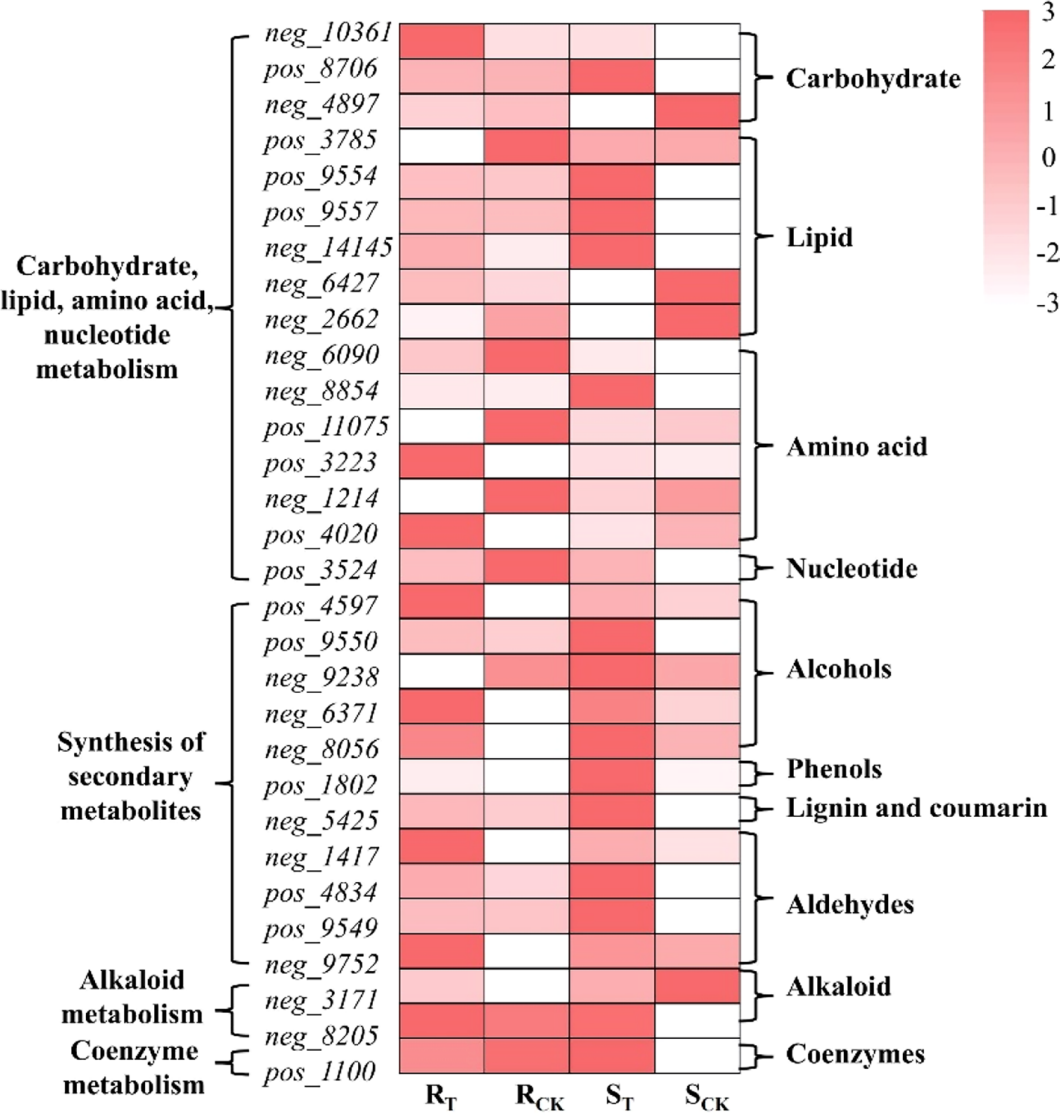
Functional classification of 30 candidate metabolites

The thirty candidate metabolites could be divided into ten categories: carbohydrates, lipids, amino acids and their derivatives, nucleotides and derivatives, alcohols, phenols, lignin and coumarins, aldehydes, alkaloids and coenzymes. Among them, there were six metabolites related to the synthesis of secondary metabolites, two for the metabolism of alkaloids and one associated with coenzyme synthesis. There were also 21 metabolites associated with carbohydrate, lipid, amino acid and nucleotide metabolism (Fig. 8).

### Metabolic pathways associated with hub genes and metabolites

**Fig. 9:**
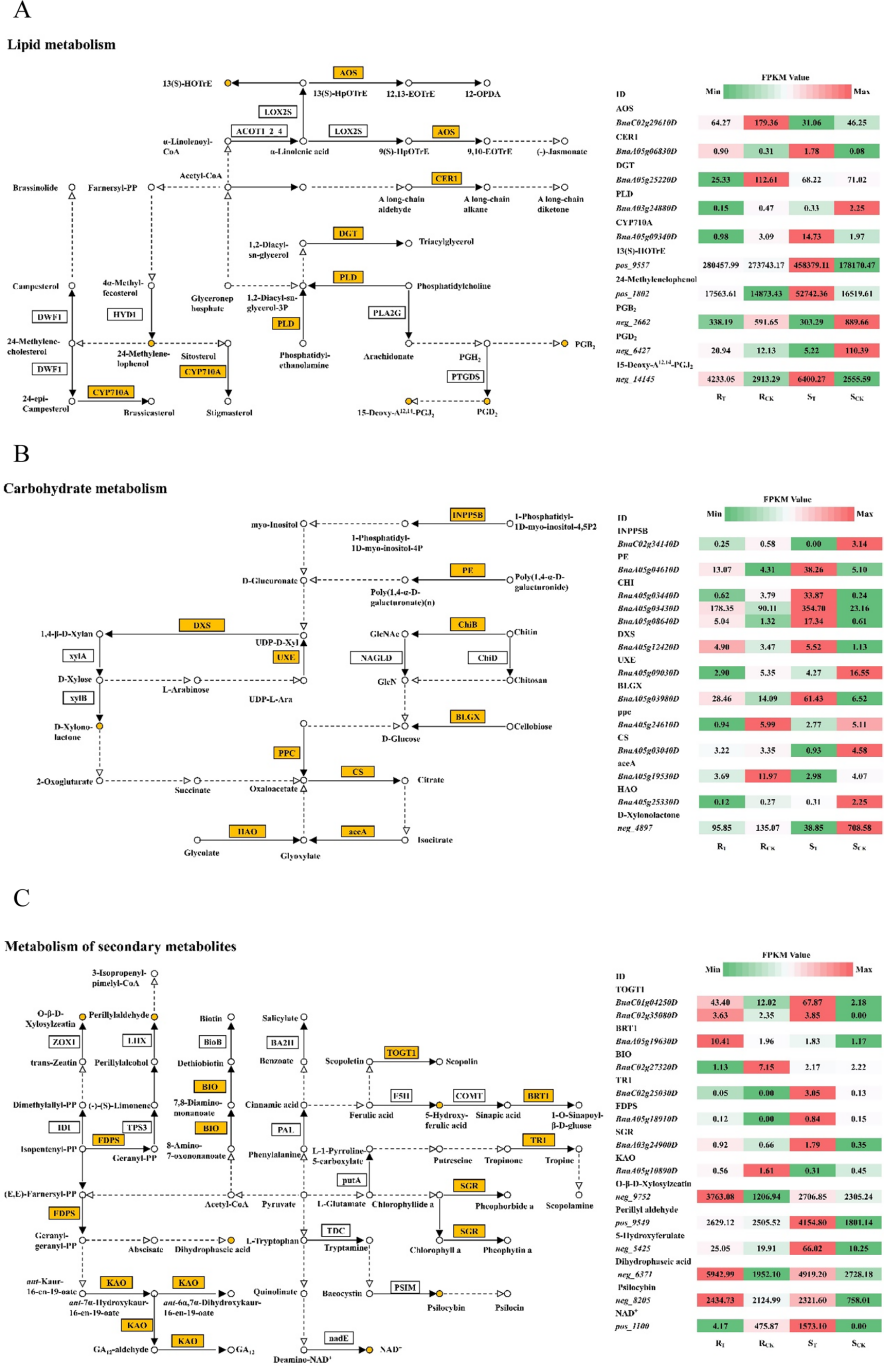
Metabolic pathway maps associated with hub genes and metabolites. **A** Lipid metabolism. **B** Carbohydrate metabolism. **C** Metabolism of secondary metabolites

KEGG metabolic pathway analysis showed that hub genes and metabolites related to aluminum stress in rapeseed roots were mainly enriched in the metabolism of lipids, carbohydrates and secondary metabolites (Fig. 9).

### Lipid metabolism

Lipids are the main components that comprise the membrane and cytoplasm of organisms and can be synthesized, stored and utilized by organisms as nutrients. Five hub genes and five differential metabolites were identified as related to lipid metabolism (Fig. 9A): *BnaA05g06830D* (aldehyde decarbonylase, *CER1*), *BnaA03g24880D* (phospholipase, *PLD*), *BnaA05g25220D* (GDSL-motif lipase 4, *DGT*), *BnaC02g29610D* (hydroperoxide dehydratase, *AOS*) and *BnaA05g09340D* (sterol 22-desaturase, *CYP710A*). In the *S* line, *CER1* was upregulated 1.12-fold and *PLD* was downregulated 1.95-fold, while in the *R* line, *CERI* was upregulated 4.16-fold and *PLD* was downregulated 2.90-fold under aluminum toxicity. *CER1* was associated with cutin, suberine and wax biosynthesis (ko00073), and the production of stem epicuticular wax, while *PLD* was involved in glycerophospholipid metabolism (ko00564) and played a role in membrane lipid modulation. Both *AOS* and *CYP710A* are members of the cytochrome P450 family, the former functioning as an allelic oxide synthase, while the latter plays an important role in growth and development, stress responses and pathogenesis. In addition, *neg_6427* (PGD_2_) and *neg_14145* (15-deoxy-Δ^12,14^-PGJ_2_) were involved in arachidonic acid metabolism(ko00590), and *pos_9557* (13(*S*)-HOTrE) was involved in *α*-linolenic acid metabolism (ko00592). The main unsaturated fatty acids are α-linolenic acid and arachidonic acid. In the *S* line, *pos_9557* was significantly increased 1.38-fold and *neg_14145* was increased 1.33-fold; *neg_6427* decreased by about 15 times in the *S* line exposed to Al^3+^ but increased by nine times in the *R* line.

### Carbohydrate metabolism

Carbohydrates are the main energy supplying substances in living cells and have important functions in regulating cell activity. Twelve hub genes and one candidate metabolite were identified in the carbohydrate metabolism pathway (Fig. 9B). Three of these genes, chitinase (*CHI*), 1,4-*β*-D-xylan synthase (*DXS*) and UDP-arabinose 4-epimerase (*UXE*), participate in amino sugar and nucleotide sugar metabolism (ko00520). Other genes such as citrate synthase (*CS*), isocitrate lyase (*aceA*) and (*S*)-2-hydroxy-acid oxidase (*HAO*) are involved in glyoxylate and dicarboxylate metabolism (ko00630), which could facilitate the accumulation of osmotic substances to initiate osmoregulation and ROS clearance. Pectinesterase (*BnaA05g04610D*) and D-xylonolactone (*neg_4897*) were found, and they are in the pentose and glucuronate interconversions pathway (ko00040). The remaining genes, *INPP5B* (inositol polyphosphate 5-phosphatase)*, BLGX* (*β*-glucosidase) and *PPC* (phosphoenolpyruvate carboxylase) are associated with phosphatidylinositol signaling (ko04070), starch and sucrose metabolism (ko00500) and pyruvate metabolism (ko00620), respectively. Among the DEGs related to carbohydrate metabolism, most of the genes in the *S* line exhibited greater changes than those in the *R* line, indicating that the *S* line was more sensitive to aluminum toxicity than the *R* line.

#### Metabolism of secondary metabolites

Plant secondary metabolites are a class of products synthesized by plants during growth and development, and containing substances responsible for cell growth, reproduction and aging. Eight hub genes and six metabolites were identified in the metabolism of secondary metabolites (Fig. 9C). Both *BnaC01g04250D* and *BnaC02g35080D* encoded scopoletin glucosyltransferase (*TOGT1*), and *BnaA05g19630D* encoded sinapate 1-glucosyltransferase (*BRT1*). These three genes and one secondary metabolite, *neg_5425* (5-hydroxyferulate), were involved in phenylpropanoid biosynthesis (ko00940). *BnaA05g18910D* encoded geranylgeranyl diphosphate synthase (*FDPS*) and *BnaA05g10890D* encoded ent-kaurenoic acid monooxygenase (*KAO*); these two genes participated in terpenoid backbone biosynthesis (ko00900) and diterpenoid biosynthesis (ko00904), respectively. *BnaA05g18910D* regulated the accumulation of O-*β*-D-xylosylzeatin (*neg_9752*) related to zeatin biosynthesis. *BnaC02g25030D* encoded tropinone reductase I (*TR1*), *BnaA03g24900D* encoded magnesium dechelatase (*SGR*), and *BnaC02g27320D* encoded bifunctional dethiobiotin synthetase (BIO), which were associated with tropane, piperidine and pyridine alkaloid biosynthesis (ko00960), porphyrin and chlorophyll metabolism (ko00860), and biotin metabolism (ko00780). The secondary metabolites, *neg_6371* (dihydrophaseic acid), *neg_8205* (psilocybin)*, pos_9549* (perillyl aldehyde) *and pos_1100* (NAD^+^) were found to be involved in carotenoid biosynthesis (ko00906), indole alkaloid biosynthesis (ko00901), limonene and pinene degradation (ko00903) and oxidative phosphorylation (ko00190), respectively.

#### Confirmation of candidate gene expression using qRT- PCR

**Fig. 10:**
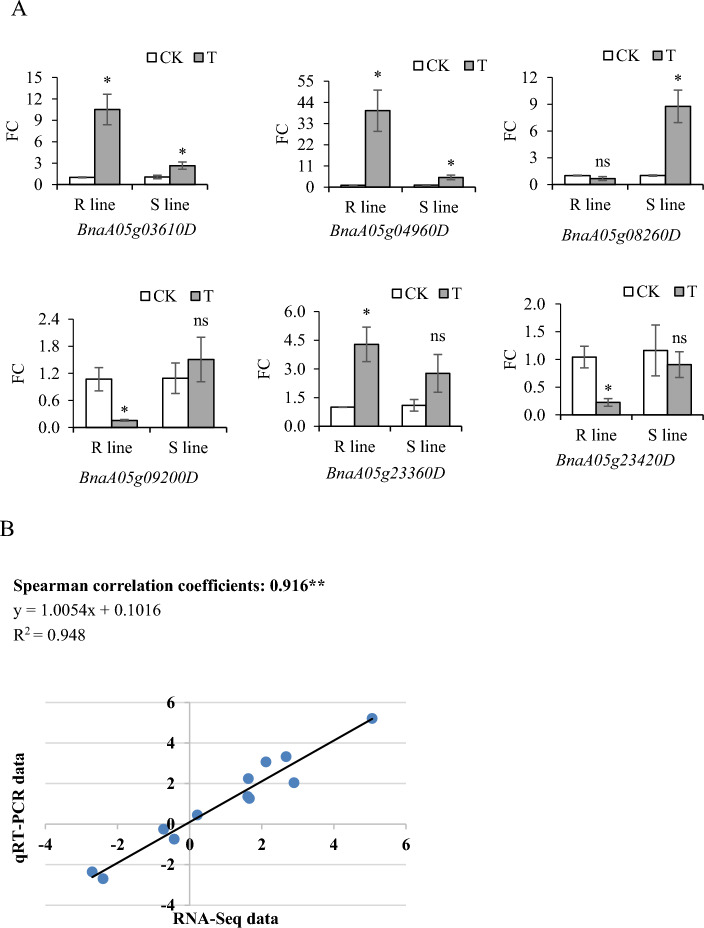
Results of qRT-PCR of candidate genes. **A** qRT-PCR of candidate genes. The error line represents the standard error of three technical repeats. Statistical significance was determined by LSD test. ns means not significantly different (*p* > 0.05); * means significantly different (*p* < 0.05). **B** Linear regression curve between qRT-PCR data and RNA-seq data. ** represents highly significant difference (*p* < 0.01)

To verify the accuracy and reproducibility of the transcriptome analysis, six candidate DEGs were selected for qRT-PCR analysis (Fig. 10A). The expression of these six genes was consistent with the RNA-Seq results in the *R* and *S* lines, and the Spearman correlation coefficient was 0.916 (*R*^2^ = 0.948) (Fig. 10B). These results demonstrated the reliability of the RNA-sequencing results.

#### Physiological indices of *R* and *S* lines under aluminum exposure

As shown in Table 3, the aluminum ion content in the roots of *S* line was higher than in *R* line. The level of POD, CAT, SOD and SP in roots of both *S* and *R* lines was significantly increased under aluminum toxicity stress (*p* < 0.01), but the increase in *S* line was less than that of *R* line. Consequently, the content of MDA in *S* line was about three times that in *R* line exposed to aluminum stress. This indicated that aluminum stress was more damaging to the cell membranes of *S* line roots.

**Table 3:** Physiological indices of rapeseed roots under aluminum stress

Physiological index	*R* line			*S* line		
	CK	Al^3+^ (µM)	Range of increase (%)	CK	Al^3+^ (µM)	Range of increase (%)
POD (U/g)	11,579.73	42,975.51	271.13^**^	17,850.81	41,710.41	133.66^**^
CAT (U/g)	133.89	216.46	61.67^**^	140.17	199.8	42.54^**^
SOD (U/g)	4,494.22	6,693.24	48.93^**^	3,516.52	4,887.42	38.98^**^
SP (mg/g)	58.45	94.67	61.97^**^	82.2	95.79	16.54^**^
MDA (nmol/g)	15.66	21.98	40.93^**^	18.45	64.25	248.27^**^
Al content (mg/g)	0.55	1.82	234.97^**^	0.55	2.16	294.67^**^

^**^p < 0.01; *POD* peroxidase; *CAT* catalase; *SOD* superoxide dismutase; *MDA* malondialdehyde; *SP* soluble protein

## Discussion

A combination of QTL localization and transcriptome analysis had been used previously to identify potential candidate genes for abiotic stress tolerance in crops (Marino et al. 2009; Wang et al. 2017). There were a large number of candidate genes in the QTL localization interval. Similarly, transcriptome analysis could identify thousands of DEGs associated with the target phenotypic traits, but these were still difficult to screen for key candidate genes related to target traits. Metabolomics could be used for qualitative and quantitative analysis of metabolites in specific tissues or cells of plants in a certain time and space. In this study, 138 key candidate genes related to aluminum stress in the roots of *Brassica napus* were identified by combining DEGs, QTGs and DAMs. Among these candidates, some are major genes that respond to aluminum stress, while the others might be chain reaction genes (minor genes). Significantly, the number of genes in each QTL interval ranged from 2 to 38 (Fig. 6). Some QTLs controlling RRL, RRD and RRT were simultaneously distributed on chromosome A05, and it was speculated that there might be major QTLs related to aluminum toxicity tolerance on that chromosome. QTL fine mapping is often considered to be one of the most important means to narrow the range of candidate genes; however, fine positioning usually requires a large crop population with heavy workload and high cost. Therefore, it is more cost-effective to combine DEGs, QTGs and DAMs to describe candidate genes associated with complex traits. We also found that some genes could affect two or more traits simultaneously (Fig. 9), which was in agreement with reports that co-mapping of QTLs for correlated traits might result from either tight linkage of multiple genes (Sandhu et al. 2001) or single gene pleiotropy (Xiao et al. 1996). The co-mapping of QTLs can help breeders identifying favorable alleles for multiple traits simultaneously in marker-assisted breeding (Liu et al. 2016).

There are two main mechanisms involved in response to aluminum stress, the aluminum exclusion mechanism and the aluminum tolerance mechanism (Singh et al. 2017), and transporters are involved in both mechanisms (Chen et al. 2022). Plants secrete organic acid anions such as malic acid through their roots, which react with Al^3+^ to form non-toxic complexes to resist aluminum stress (Yang et al. 2013). Tonoplast dicarboxylate transporter (TDT) is an important malic acid transporter that plays an important role in plant resistance to aluminum stress (Liu et al. 2017a, 2017b; Medeiros et al. 2017). In this study, expression of the gene *BnaC02g33350D* encoding TDT protein was found to be up-regulated in both the *R* and *S* lines (Table S8). Members of the ATP binding box (ABC) protein family responsible for the transfer of a variety of molecules across the plasma membrane by hydrolysis of ATP was also shown to be involved in the mechanism of aluminum resistance in plants (Ezaki et al. 2015). The ABC transporter genes, *BnaA05g07200D* and *BnaA05g23360D*, were also identified in this study. The ABC transporter OsALS1 in rice worked synergistically with the plasma membrane Al-uptake transporter, OsNrat1, to transfer aluminum ions from root cell walls into the cytoplasm where they were sequestered in vacuoles to protect plants from damage (Huang et al. 2012). Aluminum exposure upregulated lipid transporters, *BnaA03g25130D, BnaA03g215150D*, copper transporters, *BnaA03g25640D*, transmembrane proteins, *BnaA05g11040D*, and ammonium transporters, *BnaA09g00320D*. These genes could regulate and maintain cell homeostasis by influencing the transport of lipids, metal ions and penetrants.

Aluminum toxicity caused by acid soil induced reactive oxygen species (ROS), which in turn increased lipid peroxidation and protein oxidation in plants, restricted ion transport and altered membrane fluidity (Awasthi et al. 2019); however, Al also activated the oxidative stress genes, peroxidase, catalase, glutathione-*S*-transferase in plants which eliminated ROS (Chauhan et al. 2021). It was observed that SOD, POD and CAT activities were higher in the roots of *R* and *S* lines under Al stress (Table 3), but the increase in antioxidant enzyme activity in *R* line was greater than that in *S* line. MDA is an accepted indicator of membrane lipid peroxidation (Shahnaz et al. 2011) and the MDA content in *R* line was much lower than in *S* line exposed to Al stress, suggesting that increased antioxidant enzyme activity in the *R* line led to the decrease in lipid peroxidation. We also determined the expression of the redox reaction genes encoding glutathione-*S*-transferase (*BnaA05g12560D and BnaA03g26130*D) and two genes encoding cytochrome P450 (*BnaA05g04960D and BnaA05g09340D*), and all were upregulated in *R* and *S* lines in response to Al; but, the *R* line had higher expression levels than the *S* line (Table S8). RRTF1 (redox responsive transcription factor 1) retarded programmed cell death induced by salt stress through ROS inhibition (Soliman and Meyer 2019), and we found similar affects in *R* line exposed to aluminum ions. Therefore, the genes involved in redox reactions might also be important in alleviating Al^3+^ toxicity in plants.

Co-expression analysis of DEGs and DAMs is an effective way to identify the main metabolic pathways and key regulatory factors, influencing plant response mechanisms to stress. Plant hormones play an important role in stress alleviation (Liu et al. 2022), and the three metabolic pathways involved in the Al^3+^stress response in this study were mostly located upstream of genes for plant hormone synthesis (Fig. 9).The expression of the MtN21 transporter family protein, *BnaC02g24380D*, was upregulated in *S* line, but was not significantly different in *R* line. MtN21 is related to auxin transport, suggesting that Al^3+^ could interfere with polar auxin transport by up-regulating auxin transporter expression, leading to auxin accumulation in roots that inhibits root growth. During the biosynthesis of brassinosteroids (BRs), the extracellular domain of BRI1 binds BRs resulting in phosphorylation and activation of the kinase domain, which induces BR responses (Wei and Li 2016). Overproduction of BRs can inhibit root growth by altering the normal cell cycle processes in root meristem, retarding plant growth and development (González-García et al. 2011). BKI1 is a BRI1 kinase inhibitor. In this study, *BnaC02g28720D*, which encodes BKI1, was upregulated in the *R* line, while BRI1 encoded by *BnaC02g34410D* was downregulated; there was no significant difference in the *S* line. These results indicated that *R* line plants could prevent BR inhibition of root growth by inhibiting the BR signaling pathway. This might have been reflected in the differences in root length of *R* and *S* lines under Al^3+^ stress (Table 1). Application of exogenous salicylic acid increased soybean resistance to aluminum (Liu et al. 2017a, 2017b). In this study, salicylate glucose transferase 1, which was encoded by *BnaA05g03580D*, was significantly upregulated in both *R* and *S* lines. Thus, the complex network of Al^3+^-induced plant hormone changes supports the hypothesis that plant hormones are involved in the resistance to Al^3+^-induced stress damage in rapeseed plants.

Some transcription factors are involved in adaptive stress responses, activating or inhibiting the expression of stress-related genes to alter a plant’s ability to adapt to stressful environments (Zhong et al. 2012). In this study, the transcription factors NAC, MYB, ERF and DREB played important roles in rapeseed response to Al^3+^stress (Table S8). Both NAC035 (*BnaC02g34540D*) and MYB67 (*BnaA05g26450D*) were significantly upregulated in *R* line exposed to Al^3+^. Previous research suggested that the transcription factors NAC and MYB were associated with the regulation of lignification and secondary wall biosynthesis (Zhang et al. 2018; Zhao et al. 2020). Similarly, the gene *BnaA05g04610D* encoding pectinesterase was upregulated in both *R* and *S* lines under Al^3+^ stress conditions. Pectinesterase is important for the formation of the pectin cytoskeleton in plant cell walls (Jolie et al. 2010). This suggests that these genes might help to enhance aluminum resistance by promoting cell wall biosynthesis. In addition, DREB2B (*BnaA05g27930D*) was downregulated in *R* line but upregulated in *S* line in the presence of Al^3+^. AtDREB2A and AtDREB2B were strongly induced in roots by high-salt stress and in stems and roots by dehydration stress (Nakashima et al. 2000). However, how these transcription factors might influence aluminum stress responses needs further research.

In summary, the integration of QTL mapping, transcriptomics, and metabolomics can help in identifying the most important QTLs, allowing for rapid detection of the key genes and prime candidate metabolites. Using this method, a total of 138 hub genes and 30 candidate metabolites were screened, and three pathways of rapeseed root response to aluminum stress were predicted, including lipid metabolism, carbohydrate metabolism and secondary metabolite metabolism. Overall, at the gene and metabolite levels, the response of the *S* line to Al^3+^ stress was stronger than that of the *R* line. Our results provide much-needed practical guidance in understanding the molecular mechanisms of root responses to aluminum stress, and the collection of *R* and *S* lines affords valuable germplasm resources and genotypes for further improving aluminum tolerance in rapeseed.

## Supplementary Information

Below is the link to the electronic supplementary material.

Supplementary file1 (XLSX 10 KB)

Supplementary file2 (XLSX 149 KB)

Supplementary file3 (XLSX 242 KB)

Supplementary file4 (XLSX 11 KB)

Supplementary file5 (XLSX 358 KB)

Supplementary file6 (XLSX 65 KB)

Supplementary file7 (XLSX 31 KB)

Supplementary file8 (XLSX 31 KB)

## Data Availability

The additional files are available in the Figshare repository, https://doi.org/10.6084/m9.figshare.22756904. The raw sequence data are available in the NCBI Sequence Read Archive (SRA) repository (https://dataview.ncbi.nlm.nih.gov/object/PRJNA967236), and the accession number is PRJNA967236. Data will be made publicly accessible after publication of the manuscript.

## Abbreviations

QTL: Quantitative trait locus
RNA-seq: RNA-sequence
Al: Aluminum
RIL: Recombinant inbred lines
QTGs: Quantitative trait genes
DEGs: Differentially expressed genes
DAMs: Differentially accumulated metabolites
RRL: Relative root length
RRD: Relative root average diameter
RSA: Relative root surface area
RRV: Relative root volume
RRT: Relative root tips
RDW: Relative dry weight
POD: Peroxidase
CAT: Catalase
SOD: Superoxide dismutase
MDA: Malondialdehyde
SP: Soluble proteins
TBA: Thiobarbituric acid
UPLC-Q-TOF/MS: Ultra-high performance liquid chromatography-quadrupole time-of-flight mass spectrometry
